# Realising stratified psychiatry using multidimensional signatures and trajectories

**DOI:** 10.1186/s12967-016-1116-1

**Published:** 2017-01-18

**Authors:** Dan W. Joyce, Angie A. Kehagia, Derek K. Tracy, Jessica Proctor, Sukhwinder S. Shergill

**Affiliations:** 1 Cognition Schizophrenia and Imaging Laboratory, Department of Psychosis Studies, PO63, Institute of Psychiatry, Psychology and Neuroscience, King’s College London, De Crespigny Park, London, SE5 8AF UK; 2 Department of Neuroimaging, Institute of Psychiatry, Psychology and Neuroscience (PO89), King’s College London, De Crespigny Park, London, SE5 8AF UK

**Keywords:** Stratified psychiatry, Trials, Methodology, Multivariate

## Abstract

**Background:**

Stratified or personalised medicine targets treatments for groups of individuals with a disorder based on individual heterogeneity and shared factors that influence the likelihood of response. Psychiatry has traditionally defined diagnoses by constellations of co-occurring signs and symptoms that are assigned a categorical label (e.g. schizophrenia). Trial methodology in psychiatry has evaluated interventions targeted at these categorical entities, with diagnoses being equated to disorders. Recent insights into both the nosology and neurobiology of psychiatric disorder reveal that traditional categorical diagnoses cannot be equated with disorders. We argue that current quantitative methodology (1) inherits these categorical assumptions, (2) allows only for the discovery of average treatment response, (3) relies on composite outcome measures and (4) sacrifices valuable predictive information for stratified and personalised treatment in psychiatry.

**Methods and findings:**

To achieve a truly ‘stratified psychiatry’ we propose and then operationalise two necessary steps: first, a formal multi-dimensional representation of disorder definition and clinical state, and second, the similar redefinition of outcomes as multidimensional constructs that can expose within- and between-patient differences in response. We use the categorical diagnosis of schizophrenia—conceptualised as a label for heterogeneous disorders—as a means of introducing operational definitions of stratified psychiatry using principles from multivariate analysis. We demonstrate this framework by application to the Clinical Antipsychotic Trials of Intervention Effectiveness dataset, showing heterogeneity in both patient clinical states and their trajectories after treatment that are lost in the traditional categorical approach with composite outcomes. We then systematically review a decade of registered clinical trials for cognitive deficits in schizophrenia highlighting existing assumptions of categorical diagnoses and aggregate outcomes while identifying a small number of trials that could be reanalysed using our proposal.

**Conclusion:**

We describe quantitative methods for the development of a multi-dimensional model of clinical state, disorders and trajectories which practically realises stratified psychiatry. We highlight the potential for recovering existing trial data, the implications for stratified psychiatry in trial design and clinical treatment and finally, describe different kinds of probabilistic reasoning tools necessary to implement stratification.

## Background

There is a growing consensus that psychiatric disorders—defined as syndromes in categorical diagnostic systems such as the International Classification of Diseases [1] and the Diagnostic and Statistical Manual [2]—are heterogeneous in terms of aetiology, presenting psychopathology, and treatment response. Simultaneously, it is now recognized that the search for the aetiology and treatment of psychiatric disorders is not well-served when traditional categorical definitions of syndromes in the ICD-10 and DSM-5 are effectively *equated* with disorders. Instead, the emerging paradigm of *stratified psychiatry* [3] emphasises multifactorial *predictors* or *moderators* of disorders grounded in underlying neurobiology, gene/environment interactions and intermediate endophenotypes such that the final phenotypic expression does not necessarily align with the “classical” disorder specifications of e.g. schizophrenia, bipolar affective disorder and mood disorders.

This has led to the development of the Research Domain operational Criteria (RDoC) [3–8] and the roadmap for mental health research (ROAMER) [9]. These proposals reconstruct psychiatric disorders on the basis of biological mechanism and endophenotypes that describe (1) the aetiology of the disorder, (2) help identify predictors and biomarkers for the disease and/or sub-type the disease and (3) variation in response to treatment. This may help resolve the apparent dilemma experienced in everyday clinical practice, where two patients respond differently to the same intervention—one patient’s symptoms and signs improve substantially but another’s remain stubbornly unresponsive. Through the lens of stratified psychiatry, these two patients share some features, but may not necessarily have the same disorder despite a common categorical label of, for example, schizophrenia.

Recent studies examining illness features of mood disorders have shown that combinations of clinical variables predict response to selective serotonin reuptake inhibitors (SSRIs) [10–12]. In substance misuse disorders, cocaine dependence is predicted by a combination of parameters in neurocognitive measures of impulse-control [13]. In schizophrenia, antipsychotic treatment response and pathogenesis are predicted by overlapping sets of genes [14].

To constrain our scope, we necessarily focus on the single group of schizophreniform disorders. The proposals that follow are, however, equally applicable to other psychiatric disorders but the grouping of phenotypes will differ. For example, in terms of the DSM5, depressive disorders may be too coarse-grained, but persistent depressive disorder (dysthymia) and major depressive disorder may be appropriate because they share syndromic features. However, depressive episodes with psychotic or catatonic features might be better dealt with separately because of the qualitatively different presentation of these patients.

Given our scope, and to make our examples concrete, we further focus on the neurocognitive symptoms of psychotic disorders that are clinically significant for prognosis and quality of life [15, 16] and have proven notoriously difficult to treat with either cognitive or pharmacological interventions, despite over a decade of clinical trials. Beyond the obvious explanation that some of the compounds or interventions trialled do not affect the relevant neurophysiological substrates of these features of the illness, we propose that the failure stems from methodological problems inherited from broader considerations about *disorder definition* and the nature of *treatment response* in individuals recruited into these trials.

To proceed, we first consider how disorder definition can be reconceptualised using *clinical signatures* and a multi-dimensional understanding of signs and symptoms of illness which serves recruitment of patients into studies, in other words, patient stratification. Clinical signatures represent an individual’s disease state at a given time, defined along different quantified dimensions (loosely, ‘axes’ in a multidimensional space) of symptoms, signs or quantitative/qualitative measures (such as the individual’s loading or expression for a biomarker) that we define and operationalise from first principles using concepts found in multivariate statistics and pattern recognition. In the context of both clinical trials and interventions more generally, *response* is then naturally defined as a *trajectory* between clinical signatures before and after interventions for the same patient. In contrast, in the traditional analysis of trial data, response is defined a priori as an aggregate univariate *outcome* for a *group* of patients defined by their *shared* categorical disorder. We propose that with the richer multidimensional information contained in signatures, treatment response can be framed as modelling trajectories, retaining valuable information that predicts or stratifies how individual patients might respond. We then show how this proposal can be made compatible with traditional analyses by defining outcomes as a function of trajectories, rather than starting with univariate aggregate outcomes.

These principles are then applied in the context of a systematic review of a decade of trials in the treatment of neurocognitive features of schizophreniform disorders, evaluating their suitability or “readiness” for a stratified approach. We conclude with proposals for the future design and analysis of patient-level data for trials and clinical practice.

## Step one: multidimensional definition of disorder

### Moving beyond categorical diagnoses

In psychiatry, disorders have historically been defined by necessity as *syndromes* with operationalised thresholds for symptoms and signs to justify a *categorical* diagnosis. Modern classification systems such as ICD and DSM follow from the traditions of nineteenth century medicine [17]. Psychiatric disorders appear as categories in the ICD system from version 6, published in 1948 [18] with the first edition of the DSM arriving in 1952. The empirical field-trialled approach to definitions reflected in DSM-III (1980) resulted in operational criteria, which are reflected, though less prescriptively, in ICD from version 9 (in use from 1979 to 1994). The development of the ICD follows its origins as a statistical method for indexing causes of mortality and morbidity [18] whereas the DSM-IV and later, DSM-V, continue to focus on operational criteria for making diagnoses. The ICD in its current revision (version 10) continues to be hierarchical, whereas the DSM-V is more dimensional and organised around current understanding of phenotypes. Because of it’s operational, criteria-based approach, the DSM is often used for clinical and research work while outside Europe, the ICD10 is used primarily as a hospital and healthcare-provider coding system, rather than a diagnostic system per se. In either case, clinicians use categorical diagnoses as a technical shorthand, to provide treatments to patients who fit the syndromic description (i.e. given by the DSM criteria or described by the ICD narrative) and who are *presumed* to have the nominal disorder (e.g. schizophrenia, major depressive disorder). Medications are given regulatory approval if they can be shown to treat the categorical disorder [19, 20], on the basis of clinical trials, in which patients are assigned to treatment based on the same categorical disorders. We will argue that this fails patients given our contemporary understanding of the aetiology of psychiatric disorders, how we design trials and decide on outcomes.

In the mid-twentieth century, advances in psychophysics, neuroscience and the emergence of an overarching cognitive science reframed observable behaviour in the context of its proximal causes i.e. in broadly descending levels of abstraction as cognitive, neurophysiological, molecular and genetic systems. Within a classification system, the definition of categorical diagnoses is enforced by mutual exclusivity such that these categorical labels represent a ‘disorder’ [21] and the assignment of more than one of these represents comorbidity. Clinical management is then necessarily ‘mapped’ onto these categories [22] to produce clinical guidelines for treatment. This applies even when multi-axial versions of classification systems are considered.

However, equating disorders to their syndromes is demonstrably artificial. In the past decade, research on schizophrenia has benefitted from insights into genetics of specific features of the disorder; for example, variation in DTNBP1 with severity of general cognitive performance [23], polymorphism of the COMT gene in working memory [24, 25] and executive function that are further implicated in response to antipsychotic medication [24, 26, 27]. Similarly, genomic studies [28, 29] and phenotype clustering [28, 30–32] have shown that the traditional view of diagnostic categories has less utility than symptom- and sign-specific definition of disorder and illness at the individual level.

Moreover, categorical classification cannot readily accommodate the observation of shared symptoms in the face of divergence in neurodevelopment. Diagnostic categories may exhibit marked differences in neurodevelopment [33–35] but also overlap in terms of shared symptoms: psychotic features are seen in borderline personality disorder [36–39], and bipolar disorder and schizophrenia both exhibit similarities in non-verbal communication [40], affective symptoms [41], cognitive deficits [42, 43], genetic risk factors [44–46] and a broader trans-diagnostic ‘psychosis’ phenotype [47]. The observation that signs or symptoms are rarely exclusive is an inevitable stumbling block for categorical classification in psychiatry, because they are a distinct manifestation of neurobiological dysfunction whose idiosyncratic expression in an individual patient is shaped by complex environmental factors in the history of that individual’s illness. Any given sign or symptom is likely shared across categorical disorder boundaries and is rarely observed in only a single disorder. Therefore, psychiatric disorders as entities can *only* be investigated if signs and symptoms are considered as arising in “bottom up” fashion from dysfunction in neurophysiology, genetics and environment—a principle embodied in both RDoC and ROAMER. The implication for patients is then apparent: the unique cluster of signs and symptoms, their disorder *signature*, may arise from a number of interacting dysfunctional cognitive systems that further map to a number of underlying neurobiological deficits. Thus, in a group of patients, the meaningful observable variables are the state of their *signature* components rather than their a priori diagnoses according to a categorical system.

### Dimensional definitions of disorder

In recent years, dimensional definitions of disorders have been advocated [41, 48–50] such that disease differentiation, between health and pathology as well as between *different disorders* with some shared symptoms and signs [50], proceeds by comparing a given patient state (*signature*) to *prototypes* along one or more dimensions/scales. Some have noted that this approach is complicated by the absence of a “mathematical, precise resolution of what constitutes ‘sufficiently similar’ patients” [51], which in fact succinctly captures the focus of the current proposal, as it is central to and necessary for the concept of stratified psychiatry [3].

**Fig. 1:**
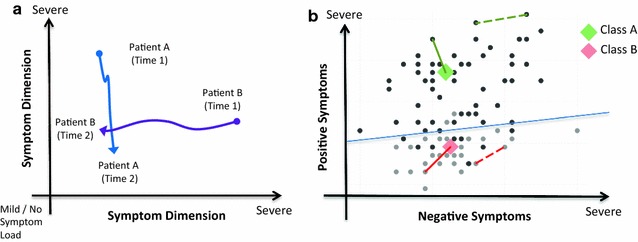
**a** Schematic representation of two patients’ signatures at time 1 (before intervention) and time 2 (after intervention) with connecting lines representing *trajectory*. **b** A space of patient signatures along dimensions of positive and negative symptoms, with *black* and *grey points* representing patients belonging to two tentative clusters of patients. *Diamonds* show the centroids (*prototypes*) of the two patient categories. *Dotted lines* represent *between* patient-signature similarity and *solid lines* represent class membership as proportional to the distance of a given patient to the nearest prototype/centroid. The *blue solid line* represents a *linear discriminant* separating the two classes such that the overall misclassification error is minimised, as might be estimated or learned by, for example, a support vector machine or linear discriminant analysis

Any proposal for a *signature* and *prototype* based approach needs to be capable of modelling differences *and* similarities between patients. For ease of exposition, but without loss of generality, consider Fig. 1a, where two perpendicular ‘axes’ form a plane (a two dimensional Cartesian space) with each axis representing a monotonically increasing scale from low-to-high symptom ‘load’ (for example, in schizophreniform illness, the axes could represent psychotic positive and negative symptom load respectively). A patient’s *signature* at a given time is defined as the point on the plane given by its two-dimensional coordinates. Figure 1a shows two patients with very different signatures at time 1, but signatures at time 2 which are close in the plane. Under this model, the cause of these different signatures and trajectories could represent (1) different interventions, (2) different responses to the same intervention or (3) patients with different disorders (but with the same categorical disorder label). We note that although we use symptoms as examples, these axes could as easily represent any quantitative state ‘marker’; for example, a genotypic risk-profile score [29], functional neuroimaging activation of cortical areas [52] or indices of neurocognitive performance [53].

When we populate the space of signatures with multiple patients, clustering of individuals in certain locations may be observed. Figure 1b expands on this showing a two dimensional example of the positive and negative symptoms scores of 100 simulated patients. The patients’ signatures are shown as two tentative clusters (black and grey points). Patients’ similarity to each other (illustrated as dotted lines) is inversely proportional to the Euclidean (‘straight line’) distance between them. This intuition of defining signatures and spaces derives from the formal definition of Hilbert and inner-product spaces that generalise Cartesian co-ordinates [54, 55] such that ideas of similarity and distance can be naturally extended to describe arbitrarily high-dimensional spaces. Each dimension can be concretely envisaged as an axis orthogonal (perpendicular) to the others and represents any measurement of clinical state. It will later be demonstrated how the 30-dimensional ‘space’ of signatures can be formed by translating the Positive and Negative Symptom Scale (PANSS) items into patient signatures.

### Operationalising signatures, prototypes and stratification

In Fig. 1b it can be seen that the two groups of patients have a *prototype*, shown as coloured diamonds that are the centroids (the multivariate statistical *means*, or *first moments*) of the two distributions and labelled Class A and B. Note that these prototypes need not represent *categorical* disorders in the ICD10/DSM5 sense, but, instead, meaningful groups of patients with sufficiently similar signatures. Stratification then becomes the task of assigning *membership* of a given patient (signature) to one of the two tentative groups (classes), where ‘membership’ can be either a discrete or continuous property.

Stratification has been implemented traditionally by inclusion and exclusion criteria that attempt to control patient heterogeneity by defining the categorical diagnosis of interest, and then specifying confounds or disease features that would alter the interpretation of the trial results. The process of specifying these criteria to arrive at a prototypical patient suitable for a given trial may serve for diseases characterised by few and narrowly defined criteria and low comorbidity, which dictates by necessity low heterogeneity among the individuals that make up that population. This however, is not the case in psychiatry, psychosis being a prime example, which demonstrates high heterogeneity, evident in well documented differences in clinical presentation as well as drug response. Traditional approaches to trial design and statistical data treatment are not equipped to deal with heterogeneity (i.e. non-normality, outliers); they use inclusion/exclusion criteria to *overcome* it, in the process sacrificing valuable data from those non-prototypical individuals that fail at the cut-off, which leads to trial outcomes and drugs that work for some but not all. In “Experiment one: patient heterogeneity and stratification” section, we illustrate how data-driven approaches are ideally suited to expose and capitalise on this heterogeneity, by taking into account the entire sample, and rather than binary inclusion/exclusion, treat each individual on the basis of a similarity score to an estimated prototype. This approach maximises the utility of each patient enrolled in a trial, or registered on a database, flexibly adapting to subtly shifting prototypes which may vary between datasets and importantly, represents a powerful tool with which to use heterogeneity rather than seek to limit it.

In the discrete case, ‘hard-classifying’ to stratify patients involves assigning their signature to one of the mutually exclusive *discrete* classes (A or B) according to a decision rule; for example, in Fig. 1b, we compute the distance of any signature to the prototypes (centroids) of Class A and Class B, then assign the signature to the class with smallest distance. Alternatively, and especially relevant to our proposal, is that membership to a class can be a *continuous* ‘soft’ property. In this case, assignment is probabilistic, i.e. a signature belongs to class A or B with probabilities 0.7 and 0.3 respectively, where the likelihood of belonging to A or B is some function of the distance. Both hard and soft membership can be made more flexible by formulating the problem in Bayesian decision theory (see [56] Chapter 2 for a thorough treatment) where the probability of class membership for a given signature can be biased towards or away from prototypes by defining more sophisticated products of a likelihood (e.g. the distance measure in this example) and priors over classes (e.g. ‘weighting’ the class membership by prevalence of certain prototypes).

### Learning models of signatures

Thus far, we have shown that multidimensional signatures can form a space, how a metric can be defined that exposes similarity or difference between patients and the uses of these definitions for ‘hard’ and ‘soft’ stratification. In any statistical model, relationships between variables are most often acquired, learned or estimated from the data. We consider two situations; first, a collection of signatures, but no a priori information about their relationship and second, a set of signatures associated with other known variables. The first scenario represents a situation where we have signatures and seek a data-driven model by inferring prototypes supported by the data. For example, given a large number of patients whose PANSS scores (a 30-item instrument) are used to populate a 30-dimensional space, we might seek a set of prototypes that represent clusters of patients that are sufficiently similar such that stratified assignment to intervention occurs by similarity in psychopathology. Of note, this represents exploration and discovery of candidate prototypes and the model is constructed by ‘unsupervised’ learning. In Fig. 1b, stratification of a new, previously unknown patient would be some function of the distance to discovered prototypes (solid green and red lines). This straightforward idea is commonplace in the literature on statistical pattern classification and machine learning—see [57] chapter 13 and 14. The second scenario represents a different situation where we have existing information about the patients, for example, where we have patient signatures before an intervention and an indicator of change following treatment (note that we do not necessarily restrict ourselves to a dichotomised or even univariate definition of response or failure, as we will show later when we consider trajectories). In this case, using existing knowledge of treatment response can help validate an existing stratification protocol or to derive one informed by known outcomes (i.e. we know which patients responded, but not *why* given their signatures). Thus, the aim is to model the ‘mapping’ from patient signatures before intervention, to treatment response after intervention. In this context, estimating the model proceeds by ‘supervised’ learning and results in a discriminant function such as the solid blue line shown in Fig. 1b where in contrast to the unsupervised case, assignment of signatures to class A and B was known in advance. Previously unseen patients are then prospectively stratified based on which ‘side’ of the discriminant line they fall.

It is worth noting that these concepts of representation, similarity/distance and learning/model estimation have a long tradition in statistics, pattern recognition and machine learning, where they appear in the context of class assignment based on prototypes by learning vector quantisation and self-organising maps [58], binary or multinomial classification using linear and quadratic discriminant analysis [59], generalised linear models such as logistic regression [60], support vector machines [61] and Gaussian process regression and classification [62].

There are important considerations in choosing any data-driven unsupervised clustering or supervised classification algorithm (when a priori assumptions about prototypes exist). For unsupervised clustering methods, the choice of similarity metric between signatures as well as the measure of cluster *optimality* will dictate the discovered prototypes and need to be justified based on the data and their intended use. Rather than use a supervised approach reflecting a priori information about *presumed* prototypes, in Experiments 1 and 2, we use unsupervised clustering to allow the data to *support* candidate prototypes that *reveal* heterogeneity. The algorithm we chose [63] embodies the aforementioned similarity metric (see “Operationalising signatures, prototypes and stratification” section) and implements an optimality measure based on a further measure that constrains the number of discovered prototypes, such that a candidate prototype differentiates itself from others while binding together similar signatures.

There have been attempts to adopt multi-dimensional principles to the problem of (1) signature definition (2) treatment outcomes and (3) diagnostic prediction. However, none has applied a multidimensional definition consistently across *all* levels from disorder definition through to disease state and outcome. For example, while multivariate signatures based on symptomatology were used in studies predicting outcome for first-episode psychosis [64] and symptom severity and persistence in post-traumatic stress disorder (PTSD) [65], only univariate and dichotomised outcomes were addressed and categorical diagnosis was presumed. When neuroimaging data were used as the signature, machine learning classifiers were used to predict *categorical* diagnosis [66], transition from an at-risk state to a dichotomised ‘psychosis versus health’ outcome [67] and dichotomised clozapine response [68, 69], or univariate aggregate predicted univariate global assessment of function (GAF) [70]. Only one study [71] used machine learning to predict multiple outcomes in major depressive disorder although again, these were dichotomised and did not model trajectories as multidimensional constructs. We argue that stratified psychiatry should avoid categorical diagnoses and univariate treatment outcomes.

## Step two: beyond univariate outcomes

Analyses of clinical trial data typically attempt to find a parsimonious group of independent variables to predict a *univariate* primary outcome, which is, most often, a continuous scalar variable representing aggregate scores on a clinical instrument or a dichotomised outcome based on a cut-off point applied to some aggregate score. For example, response to an intervention may be defined as at least 50% reduction in the overall/aggregated symptom score. Ideally, there will be predictors that exhibit low collinearity, enabling independent effects on the outcome/dependent variable to be modelled using well understood statistical methods (e.g. logistic regression or survival analysis for dichotomised outcomes, and linear/generalised linear models for continuous outcomes). The appeal is obvious—without some clear, univariate primary endpoint (dependent variable), there would appear to be no tractable way of analysing the data.

However, evidence-based treatment protocols derived from randomised controlled trials (RCT), including meta-analysed evidence and naturalistic effectiveness trials such as CATIE, do not translate reliably to clinical practice [72] and leads to publication bias [73]. This is a direct consequence of categorical disorder definition: patients rarely conform to the strict inclusion/exclusion criteria of RCTs, they often exhibit comorbidity, in itself a consequence of categorical disorder definitions as their symptoms do not conform to the demarcated boundaries of their *assumed* categorical diagnosis [74] and finally, analysis emphasises *average response* in the presumed homogenous patient group with the same categorical disorder. In recognition of this gap between the evidence base and clinical practice, researchers have begun exploring individualised/stratified medicine. Recent translational research has focused on biomarkers [75] and signatures [76, 77], where multi-dimensional clinical metrics can be used to define trajectory of illness [78], response to treatment and relapse [79]. In other words, *multivariate outcomes* derived from multidimensional signatures and their trajectories.

This problem of *average response* is shared with pain medicine, where the classification of pain syndromes is a function of diffuse patho-physiological mechanisms (which are also difficult to measure ‘objectively’) and individual response to pain medications varies substantially also as a function of psychological and social factors. A study of 200 fibromyalgia patients described treatment response to pregabalin 450 mg for 14 weeks as a bimodal distribution [80]. Given the categorical diagnosis of fibromyalgia, the notion of average response provides no useful estimate of the effect of the drug because patients tended to either respond well, or not at all.

Trial design stands to benefit from ‘responder analysis’ [81–83] which enrols patients sequentially into a trial decision-tree; failing on one “arm” of the tree for whatever reason, e.g. intolerable side-effects or failure of analgesia enrols them into an alternative treatment arm. A similar approach was used in the design of the CATIE trial [84, 85] and there is now a literature on adaptive trial design [86–88]. This should come as no surprise given the preceding discussion. Assigning patients to treatments by their categorical diagnosis, rather than by signatures or prototypes leads to loss of valuable information about individuals, and avoidable sample attrition. The definition of aggregate, univariate outcomes to measure average treatment response of the group defined by their assumed categorical diagnosis further obscures clinically meaningful treatment response. To paraphrase Moore et al. [80]—we should “expect [analgesic] failure but pursue success” by accommodating response profiles of individual patients rather than focusing on average (unimodal) response.

### Multivariate trajectories preserve response information

**Fig. 2:**
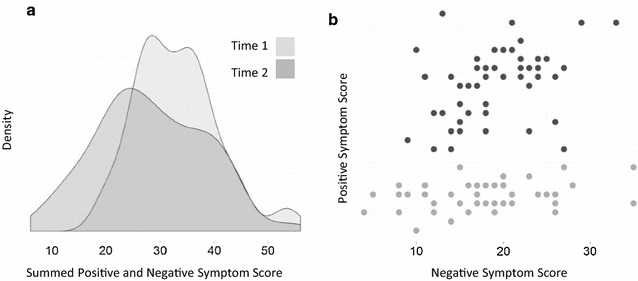
**a** Univariate distribution of the aggregate *summed total* negative and positive symptoms at time 1 (before) and time 2 (after intervention) for a simulation of 100 patients where the intervention is effective in improving *only* the positive symptoms by around 80% in approximately 50% of patients. **b** PANSS positive and negative symptom scores in two-dimensions, with *black dots* showing patients who *did not* improve (non-responders), and *light grey dots* indicating those that *did* improve (responders)

To illustrate the impact of (1) preserving multidimensional disease signatures and (2) considering multivariate trajectories and outcomes, consistent with the two outlined themes, we present the following simulated example of 100 patients and a hypothetical intervention which yields an 80% improvement for positive symptoms alone, but where only 50% of the patients respond. In assessing treatment response, we are generally interested in changes in signs and symptoms, so we retain the example of using the PANSS scale. However, changes in other relevant measures of disease states could also be used, for example, change in functional neuroimaging markers. If the usual approach of defining an aggregate, univariate outcome is adopted, such as the sum of positive and negative PANSS scores, we arrive at the distributions in Fig. 2a. Note how at time 1 (prior to the intervention, light grey) there is a clear mode to the distribution. At time 2 (following the intervention) there is a wider variance in the summed score, but the average response does not differ significantly from time 1; in other words, this appears to be a failed trial. However, in Fig. 2b which preserves the two-dimensional patient signatures in terms of positive and negative symptom load, there is a clear clustering of signatures, highlighting a group of responders (light grey) from non-responders (black). Even in a simple two dimensional signature space, a dramatic effect is revealed which is otherwise overlooked using a univariate measure of average response.

This simple demonstration illustrates the challenges faced by standard approaches to analysing treatment response based on linear (and generalised linear) models which by definition model a univariate random variable *Y* as the mean value of *Y* given the multiple predictors *X* [60]. If *Y* represents the ‘collapsed’ outcome—i.e. summed change in positive and negative scores or some dichotomised version of this, e.g. >30% change—it is unsurprising that the different response profiles which are preserved and clearly visible in the multidimensional signatures (Fig. 2b) are inaccessible and essentially lost from the analysis. The standard way of attempting to recover such effects is to use secondary outcomes, which in this case would be a change in the set of positive symptoms. The traditional treatment of secondary outcomes in the univariate framework is to correct for multiple comparisons to avoid type I errors (false positives). However, this correction may be more or less stringent, depending on the number of a priori hypothesised outcome variables, and ultimately one can never be certain that the outcome measures that survive in a particular analysis will be replicated in different datasets or future trials. The advantage of addressing changes in individual symptoms within a multivariate disease trajectory framework is that rather than assigning different status to primary and secondary outcomes, and hence variable statistical fate following correction, response is treated multi-dimensionally from the outset, so that individual symptoms are treated equally, as is their relation to their associated multidimensional disease signature.

### Operationalising trajectories

Without loss of generality we will restrict examples to two time points which can be taken to be before (time 1) and after the intervention (time 2). A signature represents a patient’s state at a given time, so a *trajectory* is a sequence of such signatures over time. A geometric interpretation of a single patient’s trajectory is the ‘line’ (a vector) in a multidimensional space connecting at least two signatures (as shown in Fig. 1a). This captures and describes change in a single patient, but provides no information about patterns, regularities or structure across many patients. Since there are potentially an infinite number of trajectories, it is necessary to find structure that enables inference over a tractable number, analogous to formally defining events and probability spaces—for discussion, see [89]. We have already shown how clusters can be learned, their prototypes defined and also, that signatures can be hard- or soft-assigned to these clusters using simple metrics. We can therefore use the finite number of prototypes to assign patients to their nearest cluster at different time points. Trajectories are then modelled as a sequence of ‘movements’ (in multidimensional space) between different clusters at each time point.

Anticipating the experiments later in this paper, assume that at time 1, our collection of signatures supports a number of clusters for which we define prototypes—e.g. as the centroid of the cluster—and assign alphabetic class labels A, B, C and so on. Applying the procedure to the same patient signatures at time 2 (post-intervention) yields a *different* number and structure of clusters which, to distinguish from time 1, we label numerically as classes 1, 2, 3 and so on. Note that these label assignments are purely categorical, and do not imply ordering, weighting or ranking. The probability that a given patient is a member of each of the classes is a function of how similar the patient’s signature is to the corresponding prototypes A, B, C at time 1. Using standard probability theory, a given patient’s trajectory is the likelihood of belonging to one of the classes (1, 2, 3) at time 2, *given* the probability that they belonged to each of the classes A, B, C, at time 1. Such a concept is captured naturally in the theory of Bayesian networks and graphical models [90] which in this example is simply the *conditional probability* of classes at time 2 on classes at time 1. This provides a tractable, countably-finite interpretation of trajectories that are supported by the data.

### Trajectories and signature spaces allow flexibility in defining outcomes

Thus far, we have used the term trajectory to refer to movements in spaces of signatures representing response to an intervention and argued that this preserves valuable information (cf. Fig. 2a, b). We have intentionally avoided the term ‘outcome’ because this implies a univariate aggregate measure that is most often dichotomised to represent success or failure of treatment.

However, in our signature and trajectory model, response can acquire the meaning of an outcome once a set of conditions over the dimensions of the signature space is applied. For example, the bottom half of the space in Fig. 1b represents signatures where there is low positive, but variable negative symptom load. Therefore, an outcome in our model is specified as a *region* in the multidimensional signature space, whose semantics can be described as ‘clinically significant change in positive, but not necessarily negative symptoms’. This can be achieved by using the discriminant line (see Fig. 1b), such that any signature, or trajectory end point, that falls in the region below the discriminant line is deemed a treatment ‘success’ in terms of specific change in positive, but not negative, symptom dimensions. Alternatively, the prototypes for classes A and B can be used to define regions based on ‘soft’ assignment within a radius around each prototype, with class B similarly capturing the meaning of treatment success. There is a clear advantage to defining an outcome as a *region* demarcated by a discriminant line (or radius around a prototype) comprising essentially multiple thresholds within a multi-dimensional signature space, instead of applying one arbitrary threshold onto a univariate, aggregate measure, e.g. total PANSS score: by modelling high-dimensional trajectories which *preserve* useful information about change, and define what is clinically meaningful multidimensionally, it becomes possible to account for heterogeneity in treatment response over individuals who respond in different ways, but respond nonetheless. In the context of a clinical trial, the inherently richer, multidimensional specification of what constitutes response, or success, effectively reduces the possibility of a false negative result, which can result from defining success according to a single point on a collapsed univariate continuum as illustrated in Fig. 2.

## Summary

1. Patients present with heterogeneous constellations of symptoms that do not respect disorder categories, even within a classical categorical diagnosis. This heterogeneity carries meaning and clinical utility. Recruitment into trials and interventions would be better defined as *targeting dimensions of disorder signs and symptoms* rather than *categories of disorder*. Variation and similarity between patients can be defined as multidimensional signature spaces.
2. The categorical assumption leads to defining *outcomes* by collapsing multi-dimensional clinical state to univariate scalar or dichotomised variables. In doing so, information is lost in favour of simplicity of analysis in trials. By representing clinical states as multidimensional spaces of signatures, data driven techniques can identify *prototypes* that identify relevant structure in these spaces. *Trajectories* defined as movement between classes and their prototypes at different times define how patients respond to interventions.
3. Aggregate or ‘collapsed’ measures of clinical state and outcomes, as well as categorical disorder definition enable measurement of only the *mean/average response* to an intervention. Therefore, the reasons why an intervention works for some individuals but not others are obscured by solely examining average response for a group. By defining multidimensional signature spaces and utilising the prototypes therein, we preserve information and can flexibly define clinically meaningful response by specifying conditions on multiple dimensions (i.e. by defining a region rather than a single threshold on a collapsed, univariate aggregate measure).

## Heterogeneity, stratification and trajectories of patients in the CATIE trial

To illustrate the potential of our multivariate framework we apply it to a real, large-scale naturalistic RCT of the efficacy of different antipsychotics. The CATIE trial [85] recruited patients on the basis of a categorical diagnosis of schizophrenia and assigned people to parallel arms for comparing the efficacy of a number of antipsychotic medications. If the originally assigned medication arm failed to treat the patient or side effects were intolerable, they were switched to another. For our experiments, following [91, 92] we extracted patient-level data with baseline PANSS scores as well as repeated neurocognitive performance measures at baseline and at 2-month follow-up (although the CATIE protocol did not specify PANSS re-evaluation at 2 months). This yielded 750 complete datasets. In Experiment 1, we use the baseline PANSS data to illustrate principles of multivariate signatures (and prototypes) yielding structure that could be used for prospective patient stratification. In Experiment 2, we apply our method to the neurocognitive data at times 1 and 2 to illustrate signatures, prototypes and trajectories.

### Experiment one: patient heterogeneity and stratification

Our hypothesis is that there is *heterogeneity* and *structure* in the 750 CATIE patients which is overshadowed by the assumptions of a categorical diagnosis of schizophrenia. By comparing our multivariate framework to univariate measures of clinical state, we show that data-driven stratification (e.g. a set of prototypes) is possible within this categorically defined group of patients.

### Methods

The PANSS naturally forms a 30-dimensional space, with variates (items) measuring 7 positive, 7 negative and 16 general symptoms. First, the 750 patients were represented in the most reductive way, as one might to measure clinical state suitable for a univariate analysis, by forming the individual *univariate* distributions of total (summed) positive, negative and general symptoms. The multivariate approach to defining the signature space was then applied. As it is impossible to visualise a 30-dimensional space, the total positive, negative and general scores were used to form *bivariate* (i.e. two-dimensional) representations of all patients, with signatures determined by combinations of positive and negative, positive and general and negative and general symptom scores, analogous to the approach in Fig. 2a, b. This representation preserves more information than the univariate approach, although there is still some loss of information as PANSS signatures form a 30-dimensional space.

Then, in the full 30-dimensional space of PANSS signatures (which cannot be plotted), we perform data-driven unsupervised clustering using Rodriguez and Laio’s algorithm [63] which finds clusters according to two criteria (using the same Euclidean metric discussed earlier) and has been shown to be robust across a number of low and high dimensional clustering problems. First, a quantity *rho* representing the local density for each patient’s signature is defined as the number of neighbouring signatures inside a specified radius. Then a quantity, *delta*, is defined for each signature as the minimum distance to any other signature with a higher local density *rho*. Prototypes are then identified as the individual signatures where *delta* is anomalously large, in other words, indicative of good separation from the nearest locally-dense clusters. We then assign each of the 750 patients to their nearest prototype in 30-dimensional space, illustrating the signature-prototype structure by colour coding each patient by their cluster membership in the bivariate representation.

### Results

**Fig. 3:**
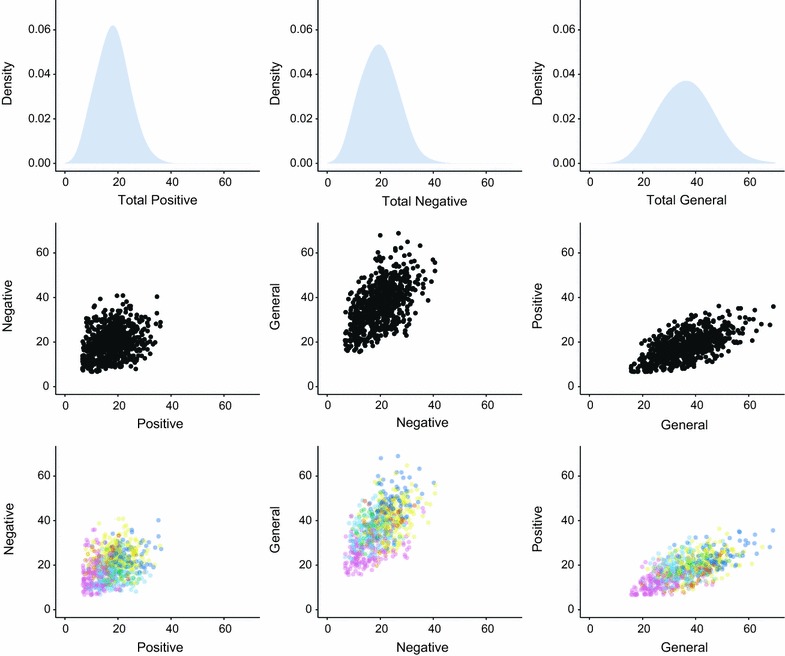
Baseline PANSS scores from the CATIE data set for 750 participants from [91, 92]. *Top Panel* distributions of univariate total positive, negative and general symptom scores across all patients. *Middle Panel* bivariate (2D) spaces of signatures obtained when each patient is represented by their positive × negative, negative × general and general × positive symptoms. *Lower Panel* the plots from the middle panel, but with each patient signature coloured according to the cluster assigned using data-driven clustering in the *original* 30-dimensional space—8 clusters were discovered in total. Data points in all plots are jittered for ease of visualisation

Figure 3 (top row) shows the univariate (single dimensional) representation for summed symptom domains (positive, negative and general). Each shows an approximately normal distribution but no clear heterogeneity beyond that captured by the mean/mode and variance.

The middle row of Fig. 3 shows bivariate plots of signatures formed by combinations of symptom domains. Note how each combination of variables shows an approximately bivariate normal distribution, but where the centroid and covariance are quite different.

Applying the clustering algorithm [63] identified 8 clusters in the full 30-dimensional space of PANSS signatures. Figure 3 (bottom row) shows the bivariate plots from the middle row, but with each signature coloured according to the cluster assigned in the *full* 30-dimensional space. It can be seen that univariate (Fig. 3, top) representations conceal heterogeneity that is visible in higher dimensional representations; as dimensionality of the representation increases, structure and heterogeneity are revealed within the signature space. In contrast to the single univariate distribution, 8 clusters emerged in the 30-dimensional space, representing an opportunity for stratification of patients by psychopathology.

### Experiment two: trajectories in neurocognitive performance

Similarly to the PANSS signatures, our hypothesis is that there is *heterogeneity* and *structure* in patients’ neurocognitive performance at baseline and follow up that can be exploited to define trajectories.

### Methods

Neurocognitive performance in the CATIE trial was defined via Z-scores that summarise performance on cognitive tasks over five domains; verbal working memory, vigilance, speed of processing, reasoning, and memory [91, 92]. This forms a 5-dimensional signature space.

We proceeded as for Experiment 1; first, the univariate distributions of each domain were computed separately, then the 5-dimensional space was formed, which can be visualised using bivariate plots of combinations of cognitive domain performance (verbal × vigilance, verbal × speed, vigilance × speed, vigilance × reasoning and so on). Prototypes were derived by applying the clustering algorithm in the 5-dimensional space, assigning each patient to the nearest cluster by distance to prototype, and finally, colour coding patients in the bivariate plots according to the assigned cluster. To illustrate trajectories, we apply the clustering algorithm separately at baseline (time 1) and two-month follow up (time 2). To show how trajectories can be formalised probabilistically, we then compute the probabilities of belonging to each cluster at time 2, given the probability of this patient being in each cluster at time 1; a simple discrete Bayesian network was modelled using the gRain package [93]. We then illustrate how a ‘test’ patient’s trajectory can be found by first computing the patient’s distance to prototypes at time 1. Then, we compute the conditional probabilities of cluster membership at time 2 given their original cluster.

### Results

**Fig. 4:**
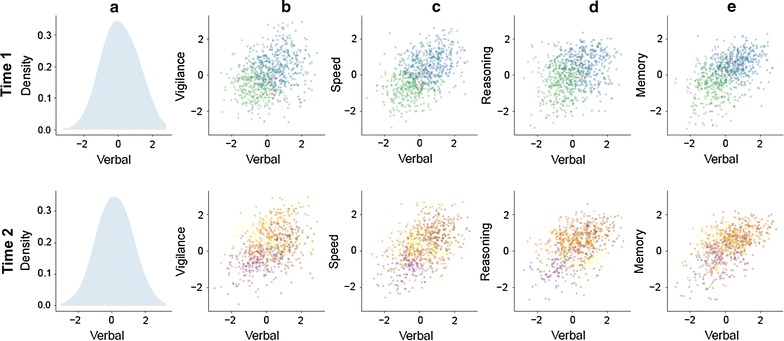
Trajectories in the CATIE neurocognitive measures with baseline and signatures prior to intervention (*Top Row*) and at 2 months after randomisation and intervention (*Bottom Row*).** a** Univariate distribution of verbal memory; **b**–**e** Bivariate plots of each combination of verbal memory against vigilance, processing speed, reasoning and working memory respectively. At *Time 1*, unsupervised clustering reveals 3 clusters (one is very small and not clearly visible) for the group. At *Time 2*, there are 5 clusters supported by the data

As the number of bivariate combinations of each of the 5 cognitive domains is large (requiring 10 separate plots), in Fig. 4, we display verbal working memory as an example known to be impaired in patients with schizophrenia and first-degree relatives [94]. Following the approach in Experiment 1, the univariate plot of verbal working memory and corresponding bivariate plots by vigilance, processing speed, reasoning and memory are shown. The top row shows time 1 (baseline) and the bottom row time 2 (follow-up at 2 months of treatment). If a univariate measure alone is defined for verbal working memory (column A) and scores at time 1 and 2 (top, bottom row) are directly compared, there is little discernible change.

The top row of Fig. 4b–e reveals 3 clusters within the patient group at time 1 supported by the data in the 5-dimensional space. Note that these are not completely contained in any part of the two dimensional representations, illustrating how information is lost as dimensionality is reduced. At time 2 (Fig. 4, bottom row, panels b–e) application of the algorithm separately on this space revealed 5 clusters, suggesting a higher degree of heterogeneity after treatment. In other words, patient trajectories can be described as ‘starting’ in one of 3 clusters and diverging to ‘arrive’ into one of a separate set of 5 clusters after treatment.

**Fig. 5:**
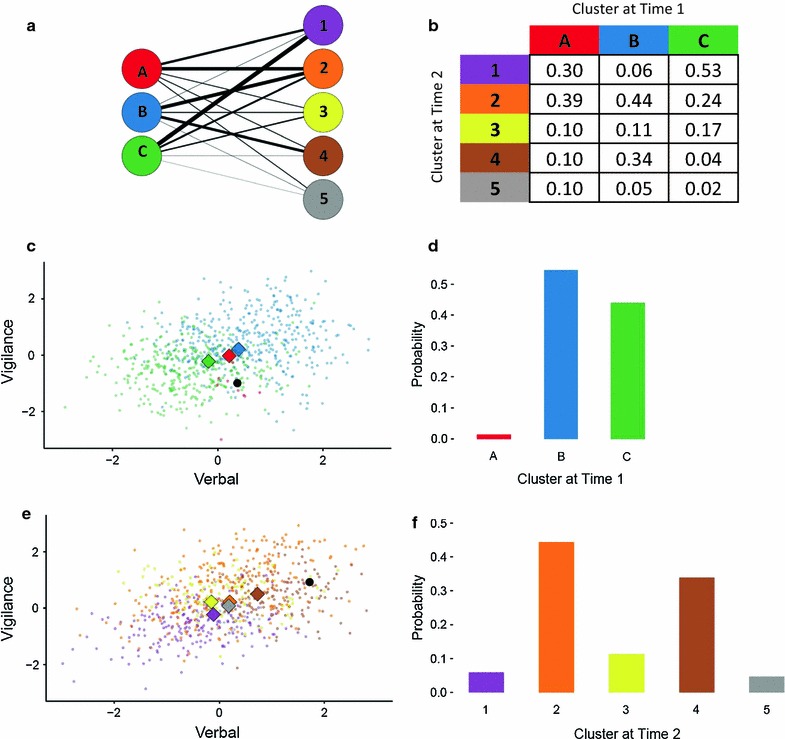
**a** Trajectories represented as a graph with nodes shown for clusters* A*,* B* and* C* at baseline (time 1) and clusters 1–5 at follow-up (time 2); heavier line weights between clusters denote higher probability of transitioning to each cluster at time 2, given the patient was in one of clusters* A*,* B* or* C* at time 1 **b** Table of probabilities corresponding to line weights in **a**. **c** Bivariate plot (Time 1) reproduced from* Column B* of Fig. 4. Baseline patient signatures at time 1, with diamonds showing approximate projected location of prototypes of clusters; a single ‘case’ patient signature is represented by a *black dot*. **d** The probability distribution of the ‘case’ patient belonging to clusters* A*,* B* or* C* (in 5 dimensional space—note that projecting into 2 dimensions results in distortion which visually misrepresents the nearest prototype). **e** At time 2, the ‘case’ patient’s signature demonstrates a trajectory of improvement in both verbal memory and vigilance performance. **f** Probability distribution of the ‘case’ patient’s membership to the clusters at time 2 *given* (conditional on) the probability the test patient was in cluster* B* at time 1

To quantify trajectories over the entire cohort of 750 patients, we computed for each patient the conditional probability of belonging to each of the five clusters at time 2, *given* the probability that the patient was in one of the three clusters at time 1. In Fig. 5, we label the three clusters at time 1 as a, b and c to emphasise that they are different to the clusters obtained at time 2 which are labelled numerically, 1–5. Figure 5a shows the overarching structure of trajectories for all 750 patients between clusters at time 1 and time 2. The line weight is proportional to the probabilities of ‘arriving’ in clusters 1 through 5 at two month follow-up, *given* the probability of starting at baseline in clusters A, B or C (Fig. 5b).

The same model can be used to *predict* a patient’s trajectory which is crucial to the idea of stratification solely on the basis of their signature at time 1. Figure 5c shows the location of a ‘case’ patient (black circle), in relation to cluster A, B and C prototypes at time 1. Figure 5d displays the likelihood at time 1 of this test patient belonging to clusters A, B or C as a probability distribution, representing ‘soft’ assignment and stratification. By querying the Bayesian network model with these likelihoods [95, 96], it is possible to extract the conditional probabilities of arriving in any of the 5 clusters at time 2. For this patient, Fig. 5f contains these corresponding predictions: clusters 2 and then 4 are the most likely ‘end points’ of the trajectory for this patient. The actual end point of this patient’s trajectory is close to the prototypes for the most-likely predicted clusters 2 and 4 (Fig. 5e).

## Systematic review of trials for neurocognitive symptoms of schizophrenia

To demonstrate the scope of our proposal and how it might influence trial design, we conducted a systematic review of the Clinical Trials registry to locate trials on cognition in schizophrenia over the period 1st January 2004 through 1st September 2015. The aim of this review was not to assess the quality of trials as reported, for example whether significant effects were obtained or whether these were adequately powered. Instead, we sought to systematically interrogate the extent to which they are characterised by assumptions of categorical and univariate assumptions relating to diagnosis and outcome, which as we have argued are incongruent with a stratified psychiatry. Using the set of criteria outlined below, which were designed in line with our proposed stratification framework, we sought to assess broadly the extent to which this recent body of work actually mirrors, conceptually and methodologically, the growing consensus for the need to stratify.

### Method and data extraction

Registered trials recruit for one or more categorical diagnoses, therefore, these definitions were necessarily used to mine the literature. Using the Clinical Trials registry (https://clinicaltrials.gov/), our search terms were as follows: trials registered between 01/01/2004 and 01/09/2015 limited to conditions defined as “schizophrenia” AND (“cognition” OR “cognitive”). Interventions were kept broad, and included trials with titles including “drugs” OR “behavioural” OR “dietary” OR “device” OR “transcranial” OR “fMRI” OR “cognitive” in order to capture the largest range of interventions. Primary or secondary outcome measures were included.

**Fig. 6:**
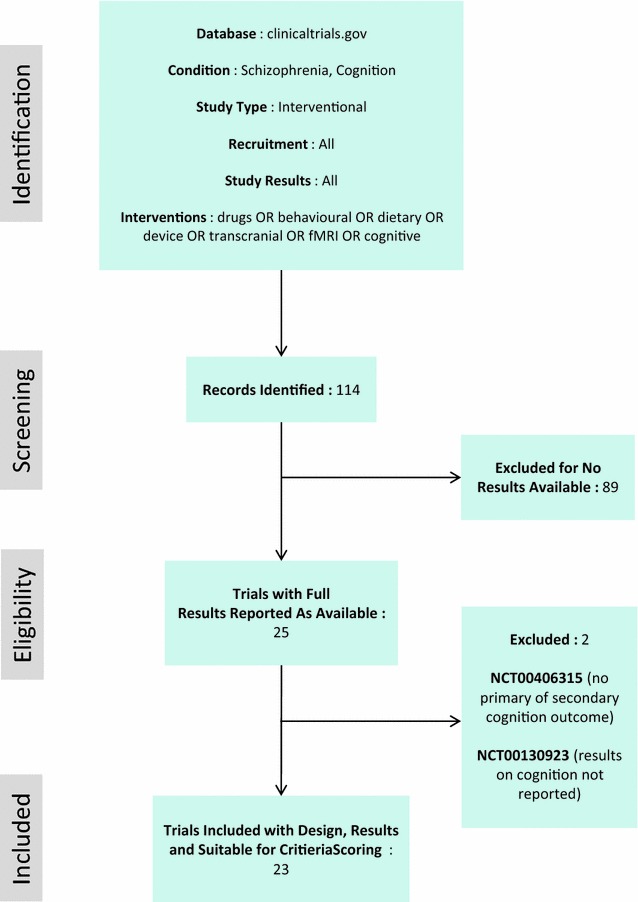
PRISMA Diagram for review of clinicaltrials.gov database

Our initial search yielded 114 trials that met the screening criteria. We then excluded 89 records where results were not accessible or were unreported. Of the remaining 25 registered trials, 2 further studies were excluded because there was no reporting of primary or secondary outcomes that included measures specific to cognition (see Preferred Reporting Items for Systematic Reviews and Meta-Analyses (PRISMA) diagram in Fig. 6) suggesting less relevance.

The design and reported analyses of the remaining 23 studies were examined. For each trial, the full text and results tables were examined by two authors (DWJ and JP). For 8/23 trials, results had also been published in journals and these articles were reviewed and tabulated alongside information from the trials database.

Each trial was assessed according to the following criteria:

1. *Diagnostic categories* (e.g. schizophrenia, schizoaffective) and the criteria used, which, for all studies, was either DSM-IV, DSM-IV-TR or not specified.
2. Within the diagnostic inclusion criteria, which *specific symptoms/features* (for example, as used in the research domain operational criteria), *domains* (e.g. positive or negative symptoms) or *smaller groups of symptoms* were specified as inclusion/exclusion criteria. This represented recognition of the heterogeneity of the categorically labelled disorder.
3. Which *patient*-*specific variables* were included in the design—for example, whether symptom signatures/profiles measured by a specific instrument scale/subscale were considered along with endophenotypes or biomarkers such as genomic data, functional magnetic resonance imaging (fMRI), electroencephalography (EEG) features, hypothesised as mediators of response.
4. *Primary and secondary outcomes* and their respective *measurement type*: if composite/aggregate measures where used such as total or mean scores, or domain specific scores on specific subscales/instruments. For example, if a cognitive battery such as Measurement and Treatment Research to Improve Cognition in Schizophrenia (MATRICS) or Brief Assessment of Cognition in Schizophrenia (BACS) was used as primary outcome, were individual sub-tests measured and reported, or only the composite “overall” score. The *measure of change* was also examined in terms of hours, days, weeks, months or years and whether *statistically significant results* were reported.
5. Whether the primary and secondary outcomes where tested relative to *patient*-*specific variables*—for example, change on immediate recall tests with respect to a given biomarker or accounting for symptom signatures or responder status.

Trials accumulated points depending on the quality of specification of the aforementioned criteria, so that a score was assigned for the following:

1. If the study included specific symptoms or groups of these (Criterion 2) *or* patient specific variables (Criterion 3), it scored 1 point. If the study included *both* Criteria 2 and 3, it scored 2 points.
2. If the study considered primary or secondary outcomes *and* change measures, where it would be possible to explore individual response, trajectories and differences (e.g. if domain specific subscales rather than only composite/total scores—see Criterion 4) the study scored a further 2 points. If only total or composite scores were reported, only a single point was scored.
3. A further 1 point was scored for Criterion 5, where outcomes were analysed accounting for specified subject-specific variables. For example, if an attempt was made to address individual patient-level factors in mediating response or change in an outcome.

The range of scores was therefore 0–5. If there was insufficient reported information (either in the trials database, or in accompanying published articles), the above rubric was conservatively applied favouring a lower score. If the design of the study recorded an intention to explore features described in the criteria (e.g. to explore genetic predictors, subgroups in psychotic disorders), but it was not mentioned further (e.g. in reported results) then the rubric was applied generously, favouring the higher score. This allowed for studies that were completed and closed, but where all results had not been analysed and published on clinicaltrials.gov.

As an example, if a study recruited subjects with schizophrenia or schizoaffective disorder with specific positive features and cognitive symptoms (such as thought disorder and impaired verbal working memory), with a hypothesised biomarker such as regional fMRI activity change in then by Criteria 2 and 3, 2 points were scored. If the same study records the a specific medication as the intervention, with primary outcome being change in the biomarker and individual symptom domains, then by Criterion 4, a further 2 points are accumulated. If secondary outcomes were changes in specific symptom profiles (e.g. thought disorder) mediated or grouped by the study biomarker, a further point would be scored by Criterion 5, resulting in a total score of 5. This would describe a study where the proposals of this paper are accounted for or there is potential to re-analyse the data using the same principles. Lower scores indicated that the studies were more vulnerable to the problems described in the themes articulated in this paper.

### Results

Table 1 shows the complete set of 23 included trials. Of these, 19 were efficacy or augmentation trials, most with a novel application of a drug: 13 of these were repurposing trials, where a drug with an existing license for a different indication was trialled for cognition in schizophrenia, 5 tested new compounds and 1 was an extension study from adult to a paediatric indication. In total, 7 of the included trials have been published (citations are given in Table 1).

**Table 1:** Tabulated results extracted from 18 eligible studies in the ClinicalTrials.gov database from January 2004 through September 2015

Study (citation)	Dx	Individual symptoms/clusters	Patient specific response Factors	Primary outcome	Measurement type	Measure subject specific	Change considered	Sig. eff.	Secondary outcome	Measurement type	Measure subject specific	Change considered	Sig. eff.	Score
NCT00548327	Scz/SczAff	None	COMT allele	fMRI BOLD signal change in DLPFC	Group (by COMT) difference	Yes—COMT status	Hours	No	Neuropsychological testing	Not specified	Not specified	Days	No	4
	Healthy control		Plasma drug level					No	PANSS	Not specified	Not specified	Days	No	
NCT00560937	Scz/SczAff	Cognitive testing score threshold	None	SANS	Total score	No	Weeks	Yes	CDSS	Mean score	No	Weeks	No	2
				BACS	Mean score	No	Weeks	No	CGI	Total score	No	Weeks	Yes	
				MATRICS	Composite score	No	Weeks	No	PANSS	Mean score	No	Weeks	NR	
NCT00611806 [97]	Scz (DSM)	Negative symptoms	COMT allele	PANSS	Total score		Weeks	No	MATRICS	Composite	No	Weeks	NR	5
		Positive symptoms	Plasma levels (of folate, B12, homocysteine)						PANSS	Positive score	Plasma levels/genotype	Weeks	No	
									PANSS	Total score	Genotype	Weeks	No	
									SANS	Total score	Genotype	Weeks	Yes	
NCT00931996	Scz (DSM)	PANSS score threshold	None	PANSS	Total score	No	Weeks	No	None					1
NCT00963924 (Cain et al. [133])	Scz/SczAff	Auditory discrimination	SANS score	MATRICS	Composite score	No	Weeks	Yes	MATRICS	Subscores	Yes	Weeks	Yes	4
		SANS score							SANS	Total score	Yes	Weeks	Yes	
NCT00488319	Scz (DSM)	None	None	Adverse event rate	Number of events	No	Years	NR	PANSS	5 factors	No	Years	Yes	2
									GAS	Total score	No	Years	NR	
									Motor domains	Individual domain scores	No	Weeks	NR	
									Memory	Domain scores	No	Weeks	NR	
									Verbal	Domain scores	No	Weeks	NR	
									Theory of mind	Total score	No	Weeks	NR	
									Cognitive	Domain scores	No	Weeks	NR	
									Sleep	Domain scores	No	Years	NR	
NCT01312272 (Davis et al.[134])	Scz (DSM)	None	None	Social cognition	Composite mean score	No	Days	No	Theory of mind	Total score	No	Days	No	2
									Empathy	Domain scores	No	Days	Yes	
									Social perception	Domain scores	No	Days	No	
									Facial affect recog.	Domain scores	No	Days	No	
									PANSS	Total score	No	Days	No	
NCT00922272	Scz (DSM)	Negative symptoms	None	SANS	Total score	No	Weeks	Yes	SANS	Response (total, dichotomised)	No	Weeks	No	3
									SANS	Remission (total, dichotomised)	No	Weeks	No	
									SANS	Domain scores	No	Weeks	Partial	
									PANSS	Domain scores	No	Weeks	Partial	
									CGI	Domain scores	No	Weeks	No	
									BACS	Total score	No	Weeks	No	
									Cognitive	Domain scores	No	Weeks	Partial	
									California performance skills	Domain scores	No	Weeks	No	
									SAS	Total score	No	Weeks	No	
									BAS/BARS	Total score	No	Weeks	No	
									Sleep	Domain scores	No	Weeks	No	
									CDSS	Total score	No	Weeks	No	
NCT00487942 (Kane et al. [135])	Scz (DSM)	None	Named SGA (antipsychotic)	MATRICS	Composite score	No	Weeks	No	MATRICS	Domain scores	No	Weeks	No	3
									Frontal Tests	Domain scores	No	Weeks	No	
									Actigraphy	Mean scores	No	Weeks	No	
									SCoRS	Total scores	No	Weeks	No	
									SANS	Total scores	No	Weeks	Yes	
									PANSS	Total scores	No	Weeks	No	
									PANSS	Positive symptoms	No	Weeks	No	
									Sleep	Domain scores	No	Weeks	No	
									SAS	Total scores	No	Weeks	No	
									BAS/BARS	Total scores	No	Weeks	No	
									CDSS	Total scores	No	Weeks	No	
NCT01363349	Scz (DSM)	Delusions score	Stable antipsychotic	MATRICS	Composite score	No	Weeks	No	Long term cognitive state	Not specified	No	Weeks	NR	2
		Hallucinations score	Not treatment resistant											
		Conceptual disorganisation												
		Persecutory thought												
NCT00575666	Scz	None	None	Cognition	Domain scores	No	Weeks	No	None					2
	SczAff			(digit span, verbal fluency, recall, frontal, attention, reaction time)										
				PANSS	Total score	No	Weeks	No						
				PANSS	Domain scores	No	Weeks	No						
				SANS	Total score	No	Weeks	No						
				CDSS	Total score	No	Weeks	No						
NCT00848484	Scz	None	Illness duration <1 year	BACS	Composite score	No	Weeks	No	CogState	Composite score	No	Weeks	No	2
									Executive function	Domain score	No	Weeks	No	
									Episodic memory	Domain score	No	Weeks	No	
									Working memory	Domain score	No	Weeks	No	
									Attention	Domain score	No	Weeks	No	
NCT00435370 (Zhang et al. [136])	Scz/schizophreniform	BPRS “psychotic” features	EEG P50 ratio	MATRICS	Composite score	No	Days	Yes	None					4
				RBANS	Total score	Yes	Days	Yes						
				RBANS	Domain scores	Yes	Days	Yes						
NCT01082588	Scz/SczAff	None	None	LDL cholesterol	Group mean	No	Weeks	No	None					2
				C-Reactive protein	Group mean	No	Weeks	No						
				MATRICS	Composite score	No	Weeks	No						
				PANSS	Total score	No	Weeks	No						
				PANSS	Domain scores	No	Weeks	No						
NCT00563706	Scz	Acute exacerbation (not defined)	None	PANSS	Total score	No	Weeks	Partial	PANSS	Domain scores	No	Weeks	NR	2
									CGI	Total score	No	Weeks	NR	
									CDSS	Total score	No	Weeks	NR	
NCT00506077 (Egan et al. [137])	Scz	Total PANSS in range 36–75	Matching Baseline BACS scores	BACS	Composite score	Partial (baseline matched)	Weeks	No	Neuropsychological testing	Domain scores	Partial (baseline matched)	Weeks	No	4
NCT00646581	Scz/SczAff	None	Stable antipsychotic	Cognition	Domain scores	No	Minutes	No	None					2
				(immediate and delayed recall, frontal, sustained attention, reaction time)										
NCT00455702 (Goff et al. [138])	Scz/SczAff	SczAff: Depressed type	Stable antipsychotic	SANS	Total score	Yes (demographics)	Hours/weeks	Yes	PANSS	Domain scores	No	Weeks	No	4
				Cognition	Composite score	No	Weeks	No	SANS	Response (dichotomoised)	No	Weeks	No	
				(Visual and verbal learning, frontal, working and episodic memory, motor)					CGI	Total score	No	Weeks	No	
									Cognition	Domain scores	No	Weeks	Partial	
									(visual and verbal learning, frontal, working and episodic memory, motor)					
NCT00505076 (Buchanan et al. [139])	Scz	Low BPRS scores for positive symptoms	Treated with SGA	MATRICS	Composite score	No	Weeks	No	UPSA	Summary Total score	No	Weeks	No	2
		Low performance on cognition battery							SCoRS	Total score	No	Weeks	No	
NCT00000371 (Goff et al. [140])	Scz (DSM)	Prominent negative symptoms	Stable antipsychotic	SANS	Total score	No	Weeks/months	No	SANS	Domain scores	Yes	Weeks/months	No	4
			Serum concentrations of d-cycloserine, glycine, serine, glutamate						PANSS		Yes	Weeks/months	No	
									SANS correlation with Serum concentrations		Yes	Weeks/months	No	
NCT00333970	Scz spectrum disorders	Neuropsych. Baseline	Demographics (not specified)	Verbal memory (CVLT)	Total score	Not reported	Weeks	No	None					2
		Illness variables (not specified)												
NCT01315002	Scz (DSM)	None	Smoking status	Antisaccade task performance	Percentage errors on task	No	Hours	Yes	None					2
	1st degree relatives		Stable antipsychotic											
			Genetic polymorphisms for cholinergic systems											
NCT01207219	Scz/SczAff (DSM)	None	None	Verbal acquisition (HKLLT)	Total correct	No	Weeks	NR	PANSS	Domain scores	None	Weeks	Not reported	0
	Schizophreniform, brief psychotic disorder, delusional disorder			Verbal retention (HKLLT)	Total correct	No	Weeks	NR						
				Working memory (digit space)	Total correct	No	Weeks	NR						
				Working memory (letter cancellation)	Composite score	No	Weeks	NR						

*NR* not reported, *ACSA* Amphetamine Cessation Symptom Assessment, *BACS* Brief Assessment of Cognition in Schizophrenia, *BAS* Barnes Akathisia Scale, *BOLD* blood oxygenation-level dependant, *BRIEF-A* behavioural rating inventory of executive function—adult version, *CDSS* Calgary Depression Scale for Schizophrenia, *CGAS* Children’s Global Assessment Scale, *CGI-I* Clinical Global Improvement Impression Scale, *CGI-S* Clinical Global Impression Severity Scale, *CPT* Continuous Performance Task, *ESS* Epworth Sleepiness Scale, *GAS* Global Assessment of Functioning Scale, *HQoL* Heinrich Quality of Life Scale, *HVLT* Hopkins Verbal Learning Test, *HVLT-R* Hopkins Verbal Learning Test—Revised, *HKLLT* Hong Kong List Learning Task, *LNS* Letter-Number Span Test, *MATRICS* Measurement and Treatment Research to Improve Cognition in Schizophrenia, *MCCB* MATRICS Consensus Cognition Battery, *PANSS* Positive and Negative Syndrome Scale, *PGIC* Patient Global Impression of Change, *PSQI* Pittsburgh Sleep Quality Index, *QLS* Quality of Life Scale, *SANS* Scale for the Assessment of Negative Symptoms, *SAS* Simpson Angus Scale, *SCoRS* Schizophrenia Cognition Rating Scale, *UPSA-B* University of California Performance-Based Skills Assessment, Brief Version, *WCST* Wisconsin Card Sorting Test

#### Scoring

By applying the scoring criteria to the 18 studies 1 study NCT00611806 [97] scored 5, the highest score, indicating highest compatibility with the criteria; 5 studies scored 4 indicating high compliance and the potential to use the methods proposed in this paper; 2 studies scored 3 indicating that the trial would be difficult to translate in our proposed framework. Scores of 2 and 1, indicating poor compatibility were achieved by 9 and 1 studies respectively, that is, half of the total reviewed (see Table 1).

We consider that those trials scoring 4 (NCT00548327, NCT00963924, NCT00435370, NCT00506077, NCT00455702) could be used in the framework we describe by reanalysis of the data.

Those scoring 3 or less would require substantial revision of design to use the framework proposed here, either because of design or data recording issues such as not including specific symptoms, or the use of only composite scores to measure patient signatures.

Of note, of the 6 trials scoring 4 or 5, five were published studies, suggesting that peer review processes are sensitive to and aligned with the principles discussed in this paper.

#### Factors affecting response

Of the 18 studies, 10 studies considered specific disorder features; of these, 6 defined the features using “threshold” definitions—e.g. cognitive impairment or PANSS exceeding a value—and the remainder used qualitative descriptors such as “acute exacerbation” and “depressive subtype”. Ten studies defined individual-specific descriptors which might predict or mediate response; three of these studies used biomarkers (EEG, fMRI or genomics)—the others defined clinical state measures (e.g. negative symptoms, clinically stable on named antipsychotic, illness duration and cognitive performance). Four studies both defined patient-specific descriptors *and* analysed the primary outcome with respect to these. Only one study considered all of these factors NCT00611806 [97].

#### Response/outcome measurement

For primary outcomes defined on clinical state (e.g. by PANSS scales), 4/18 studies considered domain scores—for example where individual components or subscales were used instead of mean, total or composite scores. Perhaps unsurprisingly, secondary outcomes reflected more attempts to understand multi-dimensional (rather than univariate) measurement of clinical state change: 10 studies included “domain scores” or some subscale-based test of the hypotheses, but only three actually analysed how the stated patient-specific factors would impact on the domains.

## Discussion and conclusions

In this paper, we have described how relying on categorical disorder definition leads to untenable assumptions about homogeneity in patient populations, impacts on the assessment of treatment and finally, coerces measurement of outcome to group averages sacrificing valuable response heterogeneity. We have shown how to operationalise proposals from the literature on dimensional definition of disorders consistent with proposals such as RDoC. To this end, we have introduced concepts of signatures, prototypes and trajectories, and operationalised these from first-principles using concepts from multivariate statistics, pattern recognition and probabilistic reasoning. Using well established data from the CATIE trial as an example, we have shown it is possible to apply these concepts for stratification, to define multidimensional trajectories and derive outcomes.

By systematically reviewing just over a decade of clinical trials registry data, we show that a majority of studies are contaminated or confounded by assumptions that are misaligned with principles of stratified psychiatry. This could explain the limited success in finding interventions that work for patients in the domain of neurocognitive symptoms in psychosis, which have been identified to be significant predictors of quality of life and functional outcome. Only a third of the studies reviewed scored 4 or 5 on our criteria, suggesting compatibility with the principles outlined in our proposal. We now consider specific implications of our proposal, highlighting limitations and future research directions.

Clinical symptomatology and neurocognitive data have been used throughout as examples in aid of developing our arguments, primarily to emphasise commonality between clinical *diagnoses*, *outcomes* and *stratification*. Our approach generalises to other kinds of data such as neuroimaging and genomics (“Dimensional definitions of disorder” and “Learning models of signatures” sections), as both symptomatology and biomarker data are amenable to such treatments and similarly subject to constraints arising from data complexity arguments that exceed the scope of this paper. While these are not trivial problems, they are now tractable with contemporary computational resources and recent advances in algorithm design. Genomics research [98, 99] has seen the application of pattern classification and regression techniques more generally [100, 101], with debate focusing on feature selection [102, 103], sparse sampling [57], the ‘curse’ of dimensionality [104] and asymptotic classification performance as the dimensionality of data increases [105].

### Implications for patient recruitment and stratified trials

We now consider the practical application of our proposals for trial design. The first and most obvious implication is to recruit patients for constellations of symptoms/signs in alignment with candidate biological mechanisms from the RDoC. As our systematic review demonstrates, almost all trials recruit for a diagnosis in expectation of a response that follows a unimodal *average* for the diagnostic group but few explore or even define outcome in terms of features of the illness.

Traditionally, if the aim is to study and treat disorganisation in psychosis [106, 107], a disorder (schizophrenia) and a cut-off measure over some constellation of symptoms (e.g. threshold over an aggregate of PANSS or SANS scores) might be defined, in the face of the known difficulties in robustly defining presumed syndrome or subtype of schizophrenia [108] or neurocognitive correlates [109]. Alternatively, in our framework, we would accept patients who display signs and symptoms clinically consistent with disorganisation syndrome regardless of a diagnosis of schizophrenia or schizoaffective disorder for instance. Similarly, in re-examining existing trial data or using *N*-of-1 trial databases, we would ignore categorical diagnosis. Next, each patient’s signature is defined by their neurocognitive performance and signs/symptoms profile (e.g. PANSS). Prototypes can be defined (1) according to clinical judgement (on groupings of patients’ signatures that clinicians agree are the most representative) or (2) using data-driven approaches (e.g. Experiment One). In both cases, visualisation and exploration of multidimensional signatures is required. Then, a ‘radius’ around these prototypes defines how any individual is ‘typical’ of, or similar to, the prototypes, enabling continuous inclusion based on distance from the prototype or if one requires ‘hard’ inclusion/exclusion, discriminant rules can be defined on the basis of prototypes. More patients can be included but each patient is assigned a ‘weight’ of class membership (with classes being defined by prototypes or discriminant surfaces); increasing study power, but at the potential risk of treatment exposure. Alternatively as in [3], one could data-mine existing populations of patients with schizophreniform illness for appropriate candidate prototypes, mirroring Experiment One.

The same approach applies to parallel-arm or switch-over designs [81, 82], including naturalistic designs such as CATIE. Assume we wish to compare the effectiveness of two treatments, and hypothesise that there may be benefit for patients with a disorganised syndrome and less so for patients with pronounced positive symptoms. We define a treatment arm for patients with a disorganised syndrome whose prototype is distinct from those with more pronounced positive symptoms. Patients can then be assigned based on similarity to prototypes for the treatment arms, or randomly; but their multidimensional signature is retained.

Second, we turn to trajectories. In contrast to traditionally defined univariate aggregate outcomes, the multidimensional approach necessitates specification of changes along each axis of the signature space to define outcomes a priori, e.g. for pre-trial registration. Signature spaces can be parcellated by discriminant surfaces or by ‘soft’ membership to clusters that divide the space into regions. These regions can be learned from pre-existing data, or specified a priori. Treatment response is embodied in a trajectory where the pre-treatment signature moves to a location after treatment that lies in some pre-specified region—i.e. one ‘side’ of a discriminant surface or within a radius of a prototype. Defining improvement over the constellation of disorganisation signs and symptoms will result in a region that is distinct from improvement in positive symptoms (which is lost if aggregate univariate outcomes are used). If assignment to treatment arms is random, treatment effectiveness can be crudely estimated as the number of patients who, after treatment, transitioned to each region given their similarity to their pre-treatment prototypes after treatment.

In terms of stratifying patients prospectively, recent proposals [110] consider using individual patients as *N*-of-1 trials, thereby rendering routine clinical practice a source of data to maximise data availability and a basis for making inferences. *N*-of-1 trials require formalisation of data collection in routine clinical practice and our framework is compatible with collecting and measuring response in a format useful for prospective stratification. A candidate patient case can be assigned to interventions based on prototypes acquired from historical information. As above, similarity to the prototype of interest (a cluster discovered in previous cases) is propagated forward into the analysis of trajectory and response. If two patients respond similarly, that is, their signatures *after* intervention are similar, but a third patient does not as their signature lies at a greater distance from the other two, then the respective signature distances preceding the intervention may represent a meaningful explanatory covariate in parsing why the third patient did not benefit; or indeed, predicting that another patient, at a similar distance to the third patient, will not respond to the intervention.

This model is not only useful for data with inherent heterogeneity. Even when the space of patient signatures suggests only a single group, recruitment and stratification is no worse off than when recruiting for categorical disorder. However, signatures and trajectories naturally translate into region-based outcomes which as discussed, render trials less prone to false negatives due to the flexibility afforded by their multidimensional definition. Thus, even within a relatively homogenous group of patients, trajectories offer a rich source of information on those who benefitted and the extent to which they did so.

### Implications for the nosology of psychiatric disorders

We have argued that recruiting individuals for a categorical diagnosis leads to incorrect assumptions about measuring how they respond to treatment. Primarily, we have proposed methods for stratifying patients, rather than redefining categories of disorder, just as RDoC emphasises disease signatures underwritten by biological processes. Specifically, [3] describes how a large collection of people with different a priori categorical disorders are aggregated together because of *shared disease features*; for example, major depressive disorder, dysthymia (mild depressive disorder) and depressive-phase bipolar disorder all share affective symptoms and signs. Then, large collections of endophenotype and biomarker data for this population are used to derive data-driven clusters for disorder classification (i.e. exposing common and distinct biological processes underpinning signs and symptoms).

### Implications for analysis of trials

We have argued that simple aggregation and dichotomisation of a multidimensional measure of clinical state (e.g. after a trial intervention) to a univariate scalar outcome discards and obscures relationships that may exist in the original multidimensional representation of clinical state as would any data dimensionality-reducing method [111]. Yet this represents a costly trade-off for ease of analysis and compatibility with established and well-understood statistical techniques; one is forced to derive a univariate outcome in order to utilise multivariable regression techniques which map multiple independent/predictor variables to a univariate dependent variable/outcome. The unfortunate cost of this convenience is loss of information in terms of statistical relationships present in the higher-fidelity multidimensional representation. Our framework is aligned with the RDoC and proposes refocusing on disorder features, signs and symptoms. We have made a case for ‘quantising’ these high-dimensional spaces by learning prototypes that represent data-driven locations in the space of patient signatures that provide meaningful structure. This converts high-dimensional signature spaces into a tractable number of locations such that trajectories can be defined as events to be modelled using probability theory and statistics. While we discussed learning or estimating models of prototypes and trajectories using a statistical interpretation, our proposal can also be viewed within non-probabilistic frameworks such as high-dimensional function approximation or within the broader framework of manifold theory [112, 113].

Future work is needed to expand on how uncertainty in outcomes can be represented in our framework. In frequentist and Bayesian statistics, effect sizes on outcomes have confidence and credible intervals respectively. While there is no theoretical obstacle, evidence derived from trials usually hinges on dichotomised outcomes for ease of implementation, e.g. as treatment protocols. It is likely that consulting tables of statistically significant regression coefficients for a single outcome will not suffice, and benefit instead from the implementation of evidential reasoning algorithms such as Bayesian networks in Experiment 2.

### Inference for discovery versus prediction

Whether used for predicting outcome or diagnosis, any statistical method including machine learning, multivariate statistics or the more familiar generalised linear models, is vulnerable to difficulties replicating results or *model selection* and *validation* on independent samples and this has hampered current research. For example, in the context of gene hunting, searching for neuroimaging markers of disease, or investigating the success or failure of an intervention, we seek an answer to one or more hypotheses while simultaneously, bolstering support for strong association or causality by controlling for confounds within the experimental design. The definition of validity in *explanatory* modelling [114] is therefore the discrepancy between the model’s prediction output with respect to the data it was fitted to. In this approach, hypotheses are tested (i.e. that the estimated regression coefficients on predictor variables are non-zero) with no *explicit* requirement that they generalise beyond the study data. Instead, replication and validation of findings proceeds by repeating the experiment (or trial), fitting similar models and retrospective comparison with the previous studies.

We contrast this to inference for *prediction* [114] and *stratification* where we are less concerned with discovery (explanation), and focus instead on how available information can be used to prospectively optimise treatment for the individual. In this case, the ‘gold standard’ for model validity is not its fit to collected data, but rather, how well the model predicts in the case of novel *independent* data sets. Our proposal thus comes with a health warning: inferences drawn from analyses such as those presented above using the CATIE trial data should *not* be used to retrospectively assert causality as inference for discovery. Instead, we should couch stratification and prediction in terms of probabilistic assignments of ‘weight’ given available information: e.g. “this patient has a 0.75 probability of a positive outcome on positive symptoms given treatment 1, and 0.60 with treatment 2 given they were close to prototype A, but not B”—see [115] for examples of similar reasoning with clinical examples. We propose that techniques suited to this task are likely to resemble efforts in the probabilistic reasoning community, such as graphical models that enable statistical structure in multivariate data to be discovered, modelled and then used for prediction [90, 96].

### Methodological challenges: model validation and replication

We have argued that multivariate signatures represent the fundamental ‘unit’ for describing patients and that assessment or measure of outcome in the form of trajectories should respect this. To evaluate the efficacy of such a stratified psychiatry, any predictive model should be validated by its’ ability to predict on *independent* samples (cf. explanatory models) and this has been lacking in over three decades of published studies (see [116] for a review). Validation on *truly* independent samples is challenging because patient-level data is either not suitably collected, or made available; of the existing studies similar to our proposed framework (reviewed in “Learning models of signatures” section), only two [64, 71] make use of independent samples and all rely on cross-validation for model validation and selection to mitigate against over-optimistic results due to over-fitting e.g. the bias-variance trade-off [57] and inductive bias [117]. Model validation is beyond the scope of this paper, but is addressed in the literature on model selection more generally [118] and criteria for accepting any model (acquired by any statistical method) can be approached from a frequentist, information theoretic or Bayesian perspective [119]. The choice of criteria varies depending on the intended use of the algorithm and the definition of ‘associational’ versus ‘causal’ [120, 121].

As we have demonstrated in the systematic review, data collected using existing clinical trial methods rarely align with the proposals we describe, or those of others [3, 110]. To partially mitigate against this problem, the field is currently constrained to within sample cross-validation [122, 123], whereby one part of a dataset is used to build a model, while the remainder is used for testing its predictive performance. Moreover, given small sample sizes of expensive biomarker data (e.g. neuroimaging and ‘omics’ data), cross-validation is performed with recourse to its logical extreme (leave-one-out, LOOCV), but replication failure is unfortunately predictable given that these subsamples are not truly independent. Robust validation requires testing against novel large datasets with adequate resolution in patient level data and compatible measures, conserved across multiple time points; in other words, organised with stratification and prediction in mind from the outset. There are, to our knowledge, no such repositories for psychiatric research. A particularly useful proposal [124] is to build repositories of not only data, but trained models which can be applied to other data sets. This requires standardisation and interfaces that allow exchange of data and implementations of algorithms in a common format. In this way, stratified psychiatry, and our proposal which speaks to it, can progress from the exploratory phase to validation and prospective testing.

### Conclusion

 We have presented a concrete proposal in response to the growing calls and clear need for the realisation of a truly stratified psychiatry. Our approach meets this need by integrating principles and methods from the mature fields of multivariate statistics and probabilistic reasoning with an evolving nosology in psychiatry. We have drawn on schizophrenia as a particularly challenging and pertinent field of research and clinical practice where treatment resistant disease continues to plague the lives of patients and defy scientists to the extent that it is often considered a separate clinical entity. The thorny problem of treatment resistance may be rendered more tractable if it is addressed in the context of movement in multidimensional signature spaces. By preserving and exploiting heterogeneity and embracing the principles of stratified psychiatry, our work can begin to productively focus on defining trajectories for subgroups of individuals with quantifiably similar signatures.
